# Worm blobs as entangled living polymers: from topological active matter to flexible soft robot collectives

**DOI:** 10.1039/d3sm00542a

**Published:** 2023-09-06

**Authors:** Antoine Deblais, K. R. Prathyusha, Rosa Sinaasappel, Harry Tuazon, Ishant Tiwari, Vishal P. Patil, M. Saad Bhamla

**Affiliations:** a van der Waals-Zeeman Institute, Institute of Physics, University of Amsterdam 1098 XH Amsterdam The Netherlands A.Deblais@uva.nl; b School of Chemical and Biomolecular Engineering, Georgia Institute of Technology Atlanta GA 30332 USA saadb@chbe.gatech.edu; c Department of Bioengineering, Stanford University Stanford CA 94305 USA

## Abstract

Recently, the study of long, slender living worms has gained attention due to their unique ability to form highly entangled physical structures, exhibiting emergent behaviors. These organisms can assemble into an active three-dimensional soft entity referred to as the “blob”, which exhibits both solid-like and liquid-like properties. This blob can respond to external stimuli such as light, to move or change shape. In this perspective article, we acknowledge the extensive and rich history of polymer physics, while illustrating how these living worms provide a fascinating experimental platform for investigating the physics of active, polymer-like entities. The combination of activity, long aspect ratio, and entanglement in these worms gives rise to a diverse range of emergent behaviors. By understanding the intricate dynamics of the worm blob, we could potentially stimulate further research into the behavior of entangled active polymers, and guide the advancement of synthetic topological active matter and bioinspired tangling soft robot collectives.

## Abstract 2

Living worms form “blobs,” active entangled physical structures with emergent properties. We show how these worms provide a fascinating platform to study active polymer physics.

## Graphical abstract

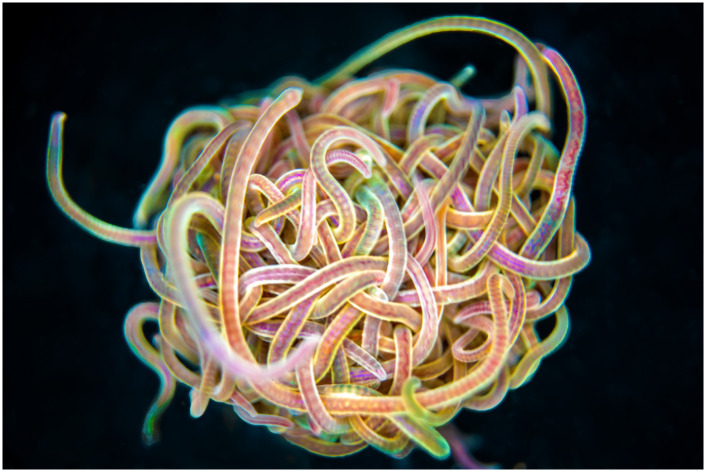

## Introduction

An essential ingredient that enables individuals to achieve more is their ability to interact with one another, ranging from transient interactions to physically entangled systems. The nature and timescale of these interactions can differ widely among both living and artificial individuals, resulting in a diverse range of complex behaviors. In general, when displaying self-repulsive interactions, individuals remain unconnected, resulting in fluid-like behavior, such as in human crowds,^1,2^ flocking birds,^3,4^ or schooling fish.^5–7^ When interactions between active individuals become attractive, large cohesive structures can form that exhibit new mechanical responses, such as elastic behavior in rafts of ants^8^ or aggregates of living cells.^9^

Within soft matter, physical entanglement governs a broad range of living and non-living systems across length scales, from birds’ nests^10^ and ant collectives^8^ to flexible polymers and filaments.^11,12^ In contrast to ordinary attractive interactions, entanglement arises from braiding and linking interactions between rod and filament-like objects, such as ant legs,^8^ sticks^10^ or polymers. Although these filamentous elements interact through repulsive contact forces, entanglement gives rise to a configurational trap, thereby generating the effective attractive forces which give entangled matter its remarkable stability.

**Fig. 1:**
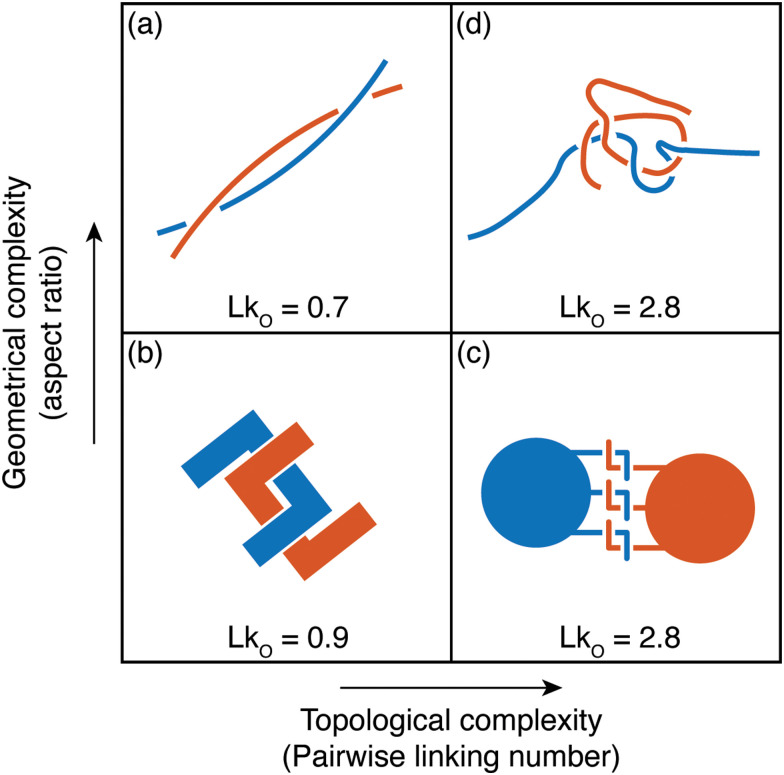
Classification of tangled matter. The components of entangled matter are classified according to their aspect ratio, and the complexity of the topological structures they can form. In each case, the linking number Lk_O_ (eqn (1)), is given for a typical entangled configuration. Lk_O_ can be thought of as a measure of the number of braids between curves. (a) and (b) The long rigid sticks (a) which make up birds’ nests,^10^ and rigid U-shaped particles^21^ (b) typically only form single braids before breaking. (c) Particles with hooks, which capture ant-like entanglement,^8^ can form multiple links despite their low aspect ratio. Here, the value of Lk_O_ shown is the total linking between all blue hooks and all red hooks. (d) Due to their flexibility and large aspect ratio, worm-like filaments are capable both of forming topologically complex structures and facilitating long-range interactions.

Owing to the remarkable diversity and ubiquity of entangled matter, classifying such systems using mathematical tools^13^ can help provide a framework in which to understand the emergent diversity in their behavior and function. For example, birds’ nests and worm blobs exhibit dramatically different material properties despite their construction as entangled collectives. One such classification scheme for entangled systems is motivated by the topological and geometrical properties of their components (Fig. 1(a–d)). Geometrical complexity, as measured by aspect ratio,^10^ distinguishes between the short-range interactions (Fig. 1(b) and (c)) of entangled ants, and the longer-range interactions mediated by more elongated filaments (Fig. 1a and d). Topological complexity can be captured using the linking number, Lk_O_, which can be defined for open curves^14,15^*γ*_1_(*s*) and *γ*_2_(*σ*), by integrating the function *Γ*(*s*,*σ*) = (*γ*_1_(*s*) − *γ*_2_(*σ*))/|*γ*_1_(*s*) − *γ*_2_(*σ*)|1
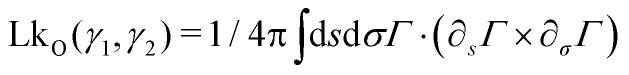
Although not strictly a topological quantity,^14,16,17^ the open linking number has emerged as a tool for quantifying such entangled interactions, separating simple interlocking (Fig. 1(a) and (b)) from more intricate intertwining (Fig. 1(c) and (d)). In passive soft matter, a classical and distinguished example of a highly topologically entangled system is a polymer solution: a liquid composed of flexible microscopic constituents with a long-aspect ratio that can form strong physical entanglements. These physical entanglements (Fig. 1(d)) give rise to the unique properties found in polymer solutions.^11,12,18–20^ Worm blobs represent an active system with similarly complex entanglements, and thus have the potential to exhibit new, functional forms of collective behavior.

At the nano- and micro-scale, biology offers numerous instances of active polymer structures, spanning from actin filaments and microtubules, which constitute the main components of the cytoskeleton in living cells,^26,27^ to flagella found in sperm, algae, bacteria, and various other planktonic microorganisms. These active systems reap the benefits of entanglement, whereby the topological entanglement of actin filaments contributes to the cytoskeleton's distinct properties. Comprehending the non-equilibrium statistical mechanics of active systems poses a significant challenge both theoretically and experimentally. Although recent advancements have been made in theory,^28–33^ there are limited synthetic experimental systems available for active filaments, which are often restricted to a small number of basic entities such as driven colloidal particles attached together,^34–36^ or self-propelled (ro)bots.^37,38^ These systems can be challenging to manipulate or access in large quantities.

Recent research has overcome these experimental challenges by utilizing active entities that rely on living biological worms: the California blackworm (*Lumbriculus variegatus*)^15,23,39^ and the sludge worms *Tubifex tubifex*.^40–42^ These studies demonstrate that the motion and dynamics of these worms can be analyzed and their activity can be easily controlled with simple methods such as temperature manipulation^23,39^ or the addition of alcohol.^40^ This makes living worms excellent candidates to investigate the behavior of active polymers in various situations.^23,39–41^ When dispersed in large quantities of water, these worms can spontaneously aggregate^41^ into highly entangled states, forming large assemblies or “blobs” that closely resemble a melt of regular polymers.^23,40^ Once entangled, the worms collaborate and exhibit vibrant, unexpected behaviors following P. W. Anderson's precept “More is different”.^43^

**Fig. 2:**
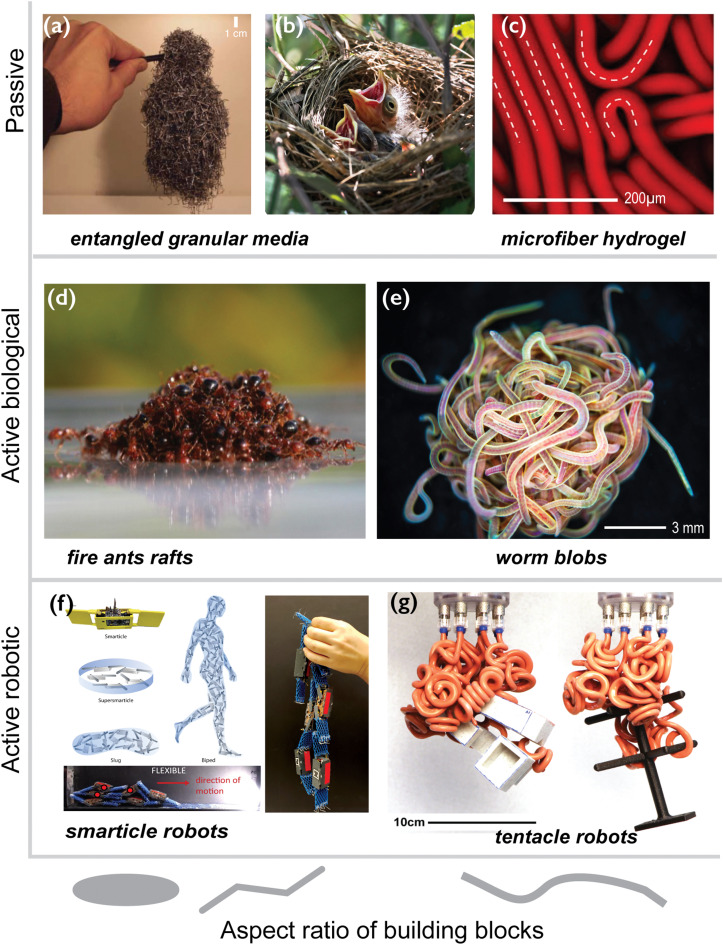
Physically and topologically entangled matter. From passive systems to active and soft robotics: in both the passive and active worlds, an increase in the particle aspect ratio leads to entanglement, and new macroscopic mechanical behaviors can emerge. (a) When increasing the aspect ratio of the particle, new physical properties can emerge as observed for a collection of staples [extracted with permission from Gravish *et al. APS*, 2023],^21^ where a (partial) degree of entanglement leads to a solid-like aggregate. (b) A bird's nest constructed from branches and twigs, a natural engineering marvel, is an example of an entangled cohesive granular structure, having remarkable properties such as plasticity despite its elastic elements and softness even though its filaments are hard. [Reproduced with permission from N. Weiner *et al.*^10^*AIP Publishing*, 2023.] (c) Irreversible formation of topological entanglements upon shear leading to a dramatic change in the rheological properties of the very-high-aspect ratio flexible fiber suspension. [Reproduced with permission from A. Perazzo *et al.*^22^*National Academy of Sciences*, 2023]. (d) In the living world, fire ants benefit from a partial degree of entanglement enabling them to build complex structures such as rafts [reproduced with permission from N. J. Mlot *et al. National Academy of Sciences*, 2023^8^] and survive a flood. (e) The worm blob exhibits a high degree of topological entanglement which is at the core of a plethora of emergent behaviors that may inspire future direction in soft robotic physics. [Reproduced with permission from V. P. Patil *et al.*^15^*AAAS*, 2023] (f) bottom: robophysical model of worm blob consist of six three-link, two-revolute joints, with planar, smart, active particles (smarticles) displaying the emergent locomotion in the collective entangled state [reproduced with permission from Y. Ozkan-Aydin *et al.*^23^*NAS*, 2023] top: an enclosed smarticle (robot) ensemble—a “supersmarticle” can self-propel diffusively using interactions arising from the shape modulation of its constituents and can also exhibit endogenous phototaxis [reproduced with permission from W. Savoie *et al.*^24^*AAAS*, 2023]. (g) Similar to how a jellyfish collects a fish, a soft robotic gripper uses the collection of active thin tentacles or filaments to entangle and ensnare objects. [Reproduced with permission from K. Becker *et al.*^25^*NAS*, 2023].

Here, we present our perspective on this new type of biological living polymer particle both as an individual entity and as a large assembly, known as the blob. We make the case that these living worms provide an outstanding experimental platform for investigating the physics of active polymer-like particles and call for a re-examination of classical polymer concepts. This perspective paper showcases the richness and emergent behaviors resulting from the combination of activity, long aspect ratio, and entanglement of the living polymer-like worms (as shown in Fig. 2). It highlights the enormous potential of this living system to achieve complex tasks autonomously, such as shape-shifting, a dream that has long been the subject of science fiction (as seen in the 1958 classic, The Blob). The worm blob opens up new possibilities and should inspire various communities, ranging from soft matter physicists to soft roboticists.

## From single individual worm to entangled worms: the blob

### Living world-inspired polymer concepts: a brief history

**Fig. 3:**
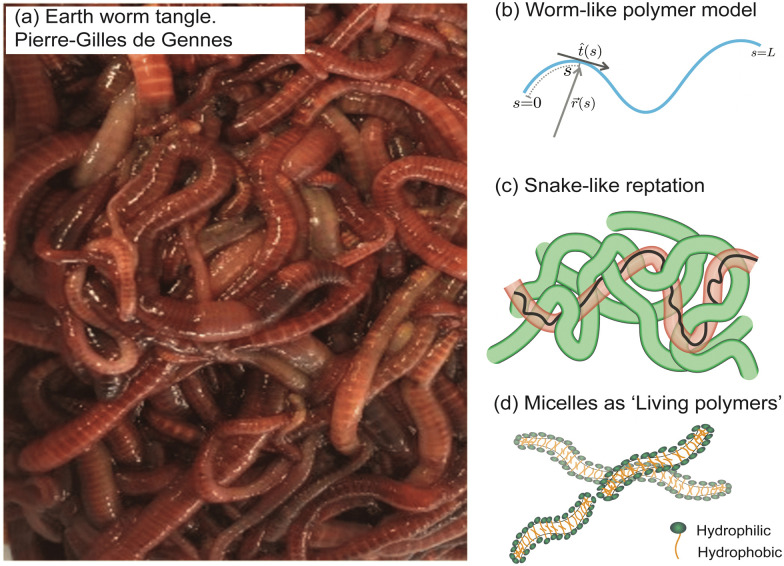
Classical concepts in polymer physics inspired by living systems. (a) Pierre-Gilles de Gennes used the analogy of a “tangle of long earthworms” to describe the motion of long polymer molecules in a melt, drawing inspiration from the movement of these worms.^44^ and image of the earthworm reproduced from (R. R. Kavle *et al.*^67^) licensed under CC BY License (MDPI, 2023). (b) The “worm-like model” represents a long and semi-flexible polymer chain of length L, parameterized by the tangent vector *t̂*(*s*). This concept was developed by Kratky and Porod (1949).^52,53^ (c) Pierre-Gilles de Gennes (1971), Masao Doi, and Sam Edwards (1978) introduced the concept of “reptation”, which describes the movement of an entangled polymer chain through a “tube” formed by adjacent chains.^11,45–47^ (d) Michael Cates (1987) developed the theoretical “living polymer” model for worm-like surfactant assemblies where the chains undergo thermally induced scission-recombination kinetics.^58,59^

This perspective focuses on the study of worms as active polymers, recognizing the extensive history where biological systems, such as worms and snakes have served as inspiration for classical polymer physics concepts. Despite the inspiration drawn from these organisms in previous work, they have primarily functioned as conceptual aids, with actual experiments or modeling being largely absent. Early advancements in polymer physics drew significant influence from these biological systems, resulting in essential theoretical models and concepts (Fig. 3). Pierre-Gilles de Gennes (Physics Nobel prize 1991), famously drew an analogy between a tangle of earthworms and the behavior of polymer melts, consisting of long, intertwined molecular chains.^44^ The “reptation theory”, which describes the unique motion of a polymer through these entangled chains as the major relaxation mechanism, was initially proposed by de Gennes in 1971^11,20^ and later expanded to the tube model by Masao Doi and Sam Edwards.^19,45–47^ This theory suggests that a polymer chain in the melt exhibits “snake-like” motion within a virtual tube formed by surrounding chains, restricting its free movement, much like snakes slithering among one another. Recently, a few groups have been investigating the effect of internal activity on the reptation of polymers, either directly,^48,49^ or by studying simulations of active polymers in porous environments.^50,51^

In 1949, Kratky and Porod^52^ described threadlike molecules as chains composed of elongated, cylindrical segments, leading to the development of a continuum “worm-like” polymer model for semi-flexible polymers.^53–55^ The model accounts for stiffness *via* the inclusion of bending elasticity and found applications in investigating a wide range of polymers, including natural biopolymers such as proteins and DNA and synthetic polymers. In the late 20th century, researchers discovered that surfactant molecules could form elongated structures resembling long polymers.^56,57^ Unlike ordinary polymers, these worm-like chains exhibit thermally induced scission and recombination dynamics on experimental time scales. Called “living polymers” due to the reversible breaking of chains at equilibrium, these structures draw inspiration from growth and division processes observed in living organisms. In the 1980s, Michael Cates became the first to integrate models of entanglement with the reversible breaking dynamics of these “living polymers”,^58–60^ spurring further research in the field.^61–66^ The study of active polymers today holds the potential to uncover new principles in non-equilibrium polymer physics and inspire the development of innovative technologies, paralleling how *passive* polymer physics has been influenced by the living world.

### The *Lumbriculus variegatus* and *Tubifex tubifex* worms: two remarkable model organisms

Belonging to the diverse phylum Annelida, both blackworms and sludge worms inhabit a wide range of freshwater environments. They play a crucial ecological role in decomposition and nutrient recycling *via* bioturbation. Remarkably, these worms can regenerate into a complete worm from each segment when cut into more than a dozen pieces, a process known architomy fission.^68^ Annelids have a long history of study, dating back to the 18th century, with a focus on their extraordinary regenerative abilities. For an extensive and detailed history of these incredible worms up to 2021, readers are encouraged to refer to the works of Rota^69^ and Martinez *et al.*^68^ Additionally, we acknowledge Charles Drewes’ passionate and inspiring contribution in proposing blackworms as a resilient and accessible organism for teaching and for research.^70–72^

Both oligochaete worms, commonly used as live food for fish, prawns, or frogs, are easily be found in pet shops. They naturally inhabit freshwater and reside in the sediment of lakes and rivers, with *T. tubifex* also found in sewer lines. Due to their unusual wiggling behavior in low-oxygen conditions, *T. tubifex* earned the nickname “the sewer creatures”: an incident in North Carolina brought attention to colonies of sludge worms (see the press article and video in Wallace^73^). Both worms have been the subject of intensive biological studies due to their ability to thrive in harsh environments, and they are commonly used as indicators of polluted environments.^74–76^

As individual entities, these worms resemble conventional polymers, characterized by long chains comprised of repeated segments that enable wiggling motion and self-propulsion, allowing the worm to crawl on a surface. Both worms measure approximately 0.3–0.5 mm in thickness and 10–50 mm in length (depending on their habitat, age, and nutrition), yielding an aspect ratio of ∼100. Their thermal random motion is negligible compared to their active motion, making them a simple model system for active polymers. Moreover, they can be cultured, harvested, and analyzed using inexpensive and simple tools, facilitating democratization of access to these systems to even budget-conscious laboratories.

Detailed analyses have been conducted on the crawling motion, dynamics, and conformations of these worms.^23,41,42^ When on a surface, *T. tubifex* exhibits a random walk with an effective diffusion constant that increases with the temperature of the surrounding environment. This has been confirmed by extracting the mean square displacement from which a long-time diffusion coefficient has been retrieved *D*_∞_ = [0.2–2] mm^2^ s^−1^.^41^ In contrast, *L. variegatus* displays more ballistic movement, provided that the ambient temperature is not excessively high.^39^

Both worms inhabit the benthic regions of bodies of water and prefer cool, dark environments. As ectothermic organisms, they primarily rely on their surrounding environment for thermoregulation. They also have photoreceptors along their tails to detect potential predators.^77^ Therefore, they exhibit both negative phototaxis and negative thermotaxis and have been observed to locomote away from these regions, individually and as a collective blob.^23^

For biological functions, these living worms may spontaneously aggregate to minimize their exposure to an excess of oxygen dissolved in water depending on their metabolic requirements.^78,79^ They form compact 3D-aggregates or highly entangled blobs, a process similar to polymer phase separation, and for which the kinetics of aggregation was recently measured.^41^ The growth occurs by the coalescence of smaller aggregates into larger ones through strong interactions – entanglement – of the individual worms. Interestingly, the coalescence between the blobs is possible because the worm blobs themselves are capable of moving. Similar to the individual motion of a worm, the blob exhibits a random walk.^41^ Surprisingly, and in stark contrast with regular polymer solutions or colloids subject to Brownian motion, measurements reveal that the diffusion coefficient of the blob is independent of its size and comparable to that of a single worm (*D*_blob_∼ 0.1 mm^2^ s^−1^).^41^ This is possible because the worms inside the entangled blob are effectively immobilized, and only the worms on the outer surface of a blob contribute forces.^15,41^

### Physics of active filaments

The worm, an elongated and slender living organism, can move its body through internal mechanisms (peristaltic motion),^80–82^ making it an ideal example of an active polymer.^39–41^ Numerous other examples of active filaments exist in the living world, with most studies focusing on motor-driven cytoskeletal filaments,^83–88^ DNA/RNA during the transcription process,^89^ cilia,^90^ flagella,^91^ sperm,^92^ rolling viruses,^93^ parasites,^94^ bacteria^95,96^ and snakes.^97^ A common feature of these systems is the interplay between activity, flexibility, and conformational degrees of freedom, which gives rise to a wide range of structural and dynamical properties at the individual polymer level^98,99^ and the collective.^29,87,100–103^

Identifying the underlying propulsive mechanisms or processes responsible for the activity of these filaments is crucial for developing computational^99,101,104–106^ and theoretical models^31,107–109^ for active polymers. Since the worms exhibit a wiggling motion and self-propelling mechanism, the closest realization might be a tangentially propelling polar active filament model. This model predicts the single active polymer dynamics^98,110–112^ and collective pattern formation exhibited by cytoskeletal filaments and bacteria.^101,106,113^

**Fig. 4:**
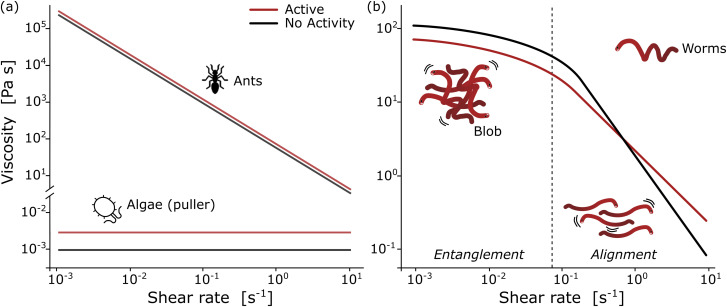
Rheological fingerprint of the blob: (a) schematic representation of the flow curves (shear viscosity as a function of shear rate) for ants^114^ and algae^115^ for two different activity states [active (red) *vs.* non-active (black)]. (b) By analogy to conventional polymers, the rheology of a blob exhibits two distinct regions at low and high deformation rates with a strong dependence on activity. Redrawn from Deblais *et al.*^40^

**Fig. 5:**
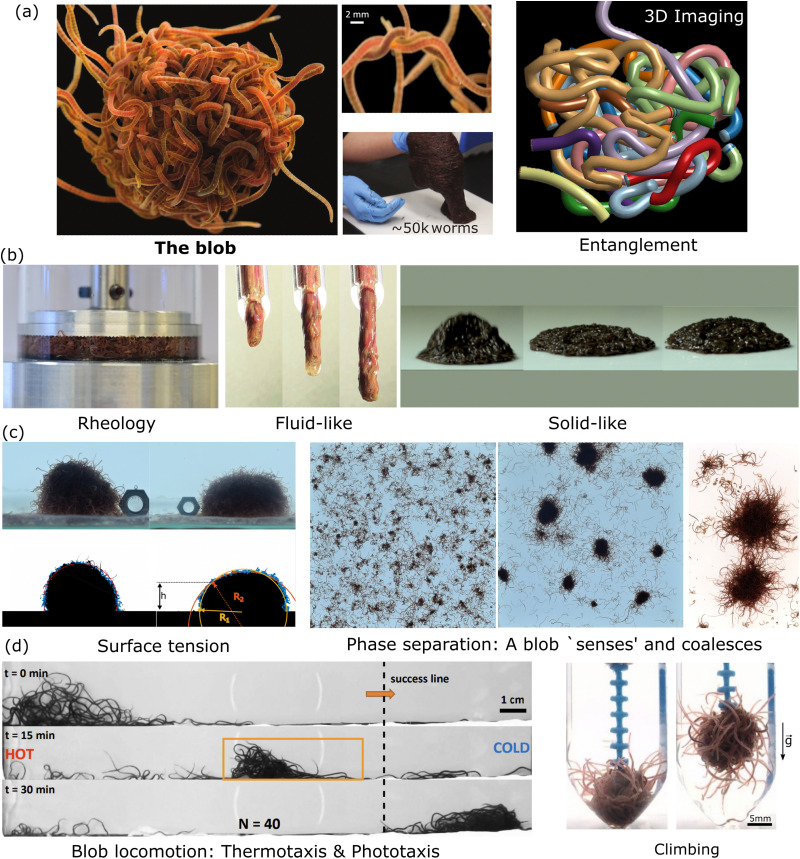
Emergent properties of a blob at a glance. (a) A blob is made of highly entangled worms as shown in the picture and reveal from 3D imaging reconstruction^15^ (Blob image credit: ©Joel Sartore/National Geographic Photo Ark, middle panel reproduced from Ozkan-Aydin *et al.*^23^ with permission from National Academy of Sciences, right panel: unpublished from VP.). (b) Mechanical/rheological properties: the blob behaves as a liquid 40 and can bounce as a solid^23^ (Left Panel reproduced from Deblais *et al.*^40^ with permission from American Physical Society, middle panel: unpublished from MSB, right panel reproduced from Ozkan-Aydin *et al.*^23^ with permission from National academy of sciences.). (c) In analogy to wetting phenomena and polymer solutions, living worms form a blob through phase separation by entanglement (reversible) and coalescence with an effective surface tension.^23,41^ (Left and Middle Panel reproduced from Deblais *et al.*^41^ with permission from American Physical Society, right panel reproduced from Ozkan-Aydin *et al.*^23^ with permission from National academy of sciences.) (d) The blob is even able to respond to external stimuli such as light, temperature,^23^ or oxygen^78,116^ to generate emergent locomotion (Left panel reproduced reproduced from Ozkan-Aydin *et al.*^23^ with permission from National academy of sciences, right panel reproduced from Tuazon *et al.*^78^ with permission from Oxford university press.).

In the collective, the worms form a blob by tangling their slender bodies with each other. Such entanglement gives intriguing rheological properties when shear is applied to the worm blob (see next section, Fig. 4).^40^ The temperature-dependent entangled network within the worm blob actively contributes to its non-Newtonian fluid behavior, enabling it to flow over long timescales (∼10 s) while preserving its solid shape during short timescales (see Fig. 5, panel (b)).^23,40^ Similarly, force measurements reveal that the worm's entanglement is oxygen-dependent.^78,116^ This highlights the presence of multiple “control knobs” that can influence the blob's behavior.

Although viscoelastic and rheological properties of active polymers have been a focus of various theoretical and computational studies,^109^ the worm blobs warrant further research owing to their resemblance to a polymer melt with complex activity patterns.

Current models of active polymers primarily focus on 2D motion, imitating the flexible living organisms that propel or glide on surfaces or interfaces. However, the formation of topologically tangled networks within 3D blobs necessitates the adoption of 3D polymer models to truly grasp their morphological dynamics. Existing theoretical work has introduced minimal models consisting of chains of active particles with specified active force directions, aiming to capture the behavior of these microscopic organisms.^39^ Although limited by its two-dimensionality and simplified self-propulsion, this model serves as a foundation for future research.

**Fig. 6:**
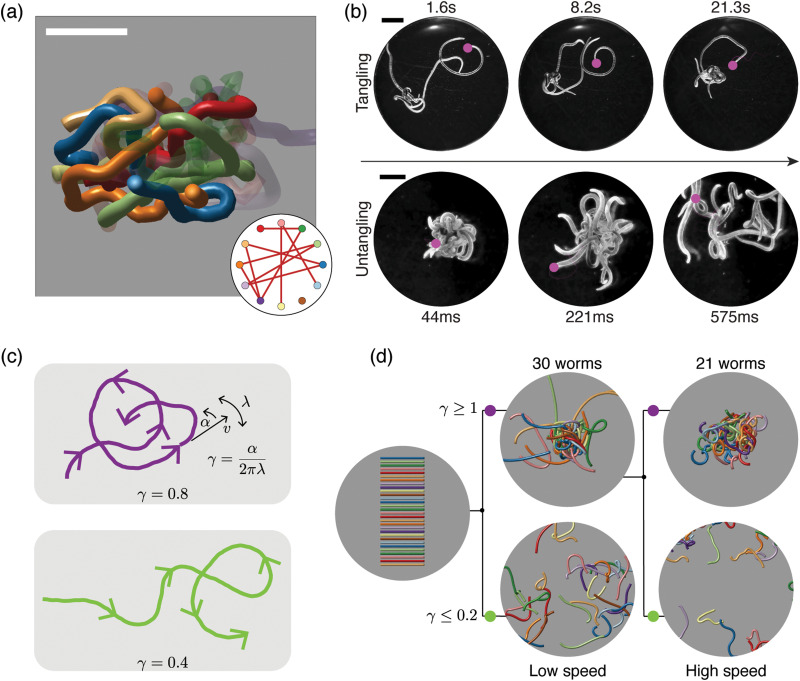
Topological structure and topological dynamics of worm blobs^15^ (a) ultrasound reconstruction of a living worm blob enables key topological interactions to be identified. Scale bar 5 mm. Inset: Tangle graph captures the topological state of the worm blob. (b) Blackworms tangle slowly (top row) but untangle rapidly, on the millisecond timescale (bottom row). The motion of worm heads (pink dots), appears to facilitate topological transitions. (c) Mean-field picture of worm tangling focuses on worm head trajectories (purple and green curves). The worm head moves at speed *v*, turns at rate *α* and switches from turning left to turning right (and *vice versa*) at rate *λ*; the worm body approximately follows the worm head. The chirality number *γ* = *α*/2π*λ* determines whether a worm head trajectory winds around obstacles more (top row) or less (bottom row). Trajectories with more winding (large *γ*) lead to tangled states. (d) Simulations of worms with different values of *γ* confirm that *γ* controls the emergent topological states, even at different worm head speeds, *v*^15^ varying *γ* leads to reversible tangle formation. Worms with large *γ* produce tangled states (top row), whereas worms with small *γ* produce untangled states (bottom row). Each worm has length 40 mm and radius 0.5 mm. (All panels reproduced from Patil *et al.*^15^ with permission from AAAS).

Recently, researchers developed a three-dimensional active stochastic model using Kirchhoff's filaments to represent worms, which effectively reproduces the tangling and untangling behaviors observed in worms (see discussion below)^15^ (see Fig. 6). Although Kirchhoff's filament model is useful in such a context, it has limitations when capturing the complex worm dynamics or simulating large-scale phenomena such as phase separation in worms due to its inability to scale adequately for large system sizes. Thus, alternative approaches are necessary to study extensive system size or phenomena at a larger spatial scale to match experimental observations.

Although hydrodynamic interactions between the filaments are known to influence the motion and coordinated movement of active polymers,^99,111^ experimental observations of both the worms (*L. variegatus* & *T. tubifex*) suggest that such interactions are negligible during blob formation^41^ This indicates a dry active polar filament model is sufficient to represent worm motion, and study the blob formation. However, it is worth noting that in aquatic ecosystems such as ponds and lakes where the worms are found, the combined effect of the bioturbation of worms and hydrodynamics is a deciding factor for sediment transport and mixing.^117–119^ Thus, to understand processes such as bioturbation, it is necessary to expand these models to encompass three dimensions and incorporate hydrodynamics.

Inspired by these worm blobs, upcoming theoretical and computational studies promise to deepen our understanding of active polymers, transcending the boundaries of conventional passive polymer physics. Such advancements hold the potential for unlocking new applications in materials science and biophysics while shedding light on the rich physics underlying these tangled active matter systems.

## Emergent properties of a worm blob

### Rheological & mechanical properties

#### Effect of activity

Inanimate materials are typically classified based on their mechanical response to external forces. Solid materials are known for exhibiting little change over time or when subjected to external forces, while liquids are strongly influenced by external stresses and can easily flow under the force of gravity. However, viscoelastic materials are much more common and exhibit complex behavior that falls between these two extremes.

Remarkable advances have been made in the study of active systems, such as dilute suspensions of pullers suspension^120^ and aggregates of fire ants,^114^ which have provided compelling evidence that activity can dramatically enhance the shear viscosity of these systems (Fig. 4(a)) compared to their inactive counterparts. For other type of system, such as pusher bacteria^121,122^ or actin filaments^123^ undergoing cycles of assembly and disassembly of bonds between active constituents, reduced shear viscosity can be observed. These findings have important implications for our understanding of the mechanical properties of materials and may lead to new approaches for the design of high-performance materials. In particular, the shear rheology tests performed on fireant aggregates^114^ have revealed a degree of shear-thinning, where the cycles of assembly and disassembly of bonds (legs) between the active constituents lead to stress relaxation. Although the viscosity of the system varies significantly over several decades, no significant effect is observed once the fire ants are rendered inactive (Fig. 4(a)). This can be attributed to the fact that in active systems like this, viscosity is primarily determined by the friction between the legs of the individuals, which highlights the importance of considering the collective behavior of active materials when designing new materials with desired mechanical properties.

The results of rheological measurements on living worms (Fig. 4(b)) provide compelling evidence of the unique and fascinating behavior of active polymer systems.^40^ Researchers were able to demonstrate a reduction in the shear-thinning behavior attributed to the activity of the worms: higher activity corresponded to a lower slope. This can be attributed to the interaction between polymer-like worms and the flow, which is more complex compared to regular polymers. The flow appears to be more efficient at orienting living polymers than conventional ones. Additionally, it has been found that the characteristic shear rate for the onset of shear-thinning is determined by the time scale of the activity (*i.e.*, characteristic time of the fluctuation of the end-to-end distance, ∼0.1 s for high activity and ∼10 s for low activity). Moreover, increasing activity levels resulted in a significant decrease in zero shear viscosity due to direct worm-worm interactions.^114^ Anomalous behavior was observed in the low shear viscosity concerning the living worm concentration for both active and low active worms.^40^ These initial results indicate a much weaker dependence on concentration, exhibiting a power law exponent of approximately 1.5, in contrast to the higher value of 3 found for regular polymers. This difference is interpreted as an intermediate value between the linear behavior observed for semi-dilute non-Brownian particle systems and an entangled polymer. These results demonstrate the rich and complex behavior of active polymerlike materials and provide important insights into the underlying physics governing their rheological properties and invite further scrutiny to elaborate suitable models.

#### Effect of oxygen concentration

Another interesting aspect of the worm blob is its changing stiffness and shape as a function of the dissolved oxygen (DO) present in the water. As aerobically respiring organisms, the oxygen consumption rate is another key parameter that worm blobs must balance along with their level of entanglement. It was previously observed that a 1 g blob of blackworms (∼150 individuals) can consume enough oxygen in a 20 mL volume of water to create an anoxic (<1 mg L^−1^) environment for themselves in around 30 minutes.^78^ As a result, the individual worms wave their tails around away from the blob to supplement their oxygen supply. This in turn lowers the structural rigidity of the blob for low DO concentration.^78^

Subsequently, a recent work measured the change in the tensile strength of the blob as a function of DO concentration.^116^ Due to the worms' waving their tail in low oxygen environments, it has been found that the blob could exert a tensile force of almost 3 times larger in magnitude when the DO concentration was high >8 mg L^−1^) compared to anoxic conditions (<1 mg L^−1^). Furthermore, it was observed that blobs of worms in ample oxygen behave like rigid objects which can topple over, while their counterparts in anoxic environments behave more like a viscoelastic gel. Therefore, blackworm blobs resemble active entangled matter whose stiffness is tunable by exogenous stimuli.

### Emergent locomotion

Worms within the blob can intertwine to form braided chains and pull together.^23^ Experiments measuring the pulling force exerted by individual worms showed that a small number of worms could generate sufficient traction force to move the collective away from negative stimulus. Additionally, locomotion in small blobs can emerge through the differential activities of individual worms at the front (puller worms generating forward thrust) and rear (wiggler worms reducing friction). These results were further validated through robophysical models using multiple three-link phototactic smarticles.^23^ The collective locomotion of worm blobs displays emergent behaviors that go beyond binary gait differentiation into wiggler and puller worms.

Building upon the previous experimental study, a model using the physics of active, semi-flexible polymers was developed.^39^ The model simulates worms as self-propelled Brownian polymers, focusing on the parameter space of aspect ratio, bending rigidity, activity, and temperature. Simulated single worms display persistent directed motion at low temperatures, while multiple simulated worms can aggregate into a blob, held together by attractive forces. The study finds that the blob can collectively navigate along a temperature gradient, provided that the tangential force and attachment strength are balanced. This yields a tradeoff between worm velocity and blob cohesiveness, with an “optimal” regime identified for effective collective locomotion in the form of a phase diagram.^39^

Perhaps even more interestingly, the worm blob as a whole can break its structural symmetry by asymmetrically changing the functional roles of individual worms in the blob and consequently, locomote across a substrate either spontaneously, or in response to chemicals, light, temperature, and fluidic gradients (see Fig. 5(d)). The modes of locomotion of the blob can be of different natures depending on the external stimulus. When the worm blob is placed in a temperature gradient, a crawling motion is observed. In this scenario, worms facing the colder temperature extend outside the blob and pull on it, while the worms experiencing the hotter temperatures on the other side wiggle their bodies to reduce friction underneath the blob, facilitating its transport.^23,39^

Thus, the blobs can crawl, form floating structures^124^ and even shape-shift to undergo complex topological transformations from pancakes to spheres when trying to avoid desiccation.^23^ Although Fig. 5 highlights that many complex behaviors are possible, many other complex behaviors are yet to be discovered that may further depend on the blob size, the activity, the type of external stimulus or the substrate properties.

### Reversible tangle topology

Due to their ability to form and escape from tangled states, worm blobs represent a model system in which to explore questions of topological dynamics of tangled filaments.^15^ A fundamental question that must be addressed before analyzing dynamics concerns classifying and quantifying the topological state of a tangle. Ultrasound imaging methods, which enable the reconstruction of the internal, 3D structure of a worm blob (Fig. 6(a)), provide insight into this problem. These datasets reveal that worm blobs are strongly interacting systems; each worm touches almost every other worm. Specific key interactions can further be selected on topological grounds. For example, pairs of worms that touch each other do not interact as strongly as pairs of worms that both touch and intertwine. Using the linking number of open curves,^14,15^ a quasi-topological quantity, this observation can be made more precise. The topological state of the worm blob may then be approximated by a tangle graph (Fig. 6(a), inset), where an edge is drawn between two nodes if the corresponding worms both touch and are sufficiently intertwined.

The topological transitions performed by worm blobs appear to be facilitated by the dynamics and trajectories of individual worms (Fig. 6(b)). Although the dynamics of worms moving in disordered tangles is complex, it can be approximated and understood using a mean-field theory (Fig. 6(c)), in which the head of each worm moves in 2D. Strikingly, a simplified mean-field picture, in which worm dynamics is approximated by a small number of parameters governing worm-head motion, is sufficient to explain tangling and untangling.^15^ In particular, consider a worm head that moves at a constant speed *v*, turns at a constant rate *α*, and switches from turning left to turning right (and *vice versa*) at rate *λ* (Fig. 6(c)). Assume further that the worm body approximately follows the worm head. Intuitively, trajectories with large *α* and small *λ* will form more loops of the same handedness before switching turning direction (Fig. 6(c); top row, purple curve). Such trajectories lead to tangled states, whereas trajectories that switch direction more often avoid one-way winding (Fig. 6c; bottom row, green curve) and lead to untangled states. The amount of winding is captured by the chirality number, *γ* = *α*/2π*λ*, a dimensionless number that plays a key role in the topological dynamics of worms. The chirality number relates the rate at which the worm head turns, *α*, with the rate at which the worm switches from turning left (right) to turning right (left), *λ*. Trajectories with large *γ* (Fig. 6(c), top row) wind more than those with small *γ* (Fig. 6(c), bottom row). The chirality number, therefore provides a mechanism for controlling the emergent topology of a worm collective.^15^

Multi-filament 3D numerical simulations of elastic filaments confirm that the chirality of worm trajectories determines the emergent topological state formed (Fig. 6(d)). Worms with large *γ* wind more and tangle, whereas worms with small *γ* untangle. Reversible tangling can therefore be achieved by varying *γ* (Fig. 6(d)), thus establishing a connection between dynamics and topology.^15^ However, the nature of this mapping between dynamics and topology raises further theoretical questions. For example, the precise mathematical relationship between *γ* in the 2D mean-field picture (Fig. 6(c)) and the open linking number between 3D worms (Fig. 6(d)) is unclear. More generally, the statistical mechanics of the transition from untangled to tangled states as *γ* increases presents an interesting avenue for future research.

## Discussion and perspectives

### Topologically entangled living polymer physics

Our recent exploration of semi-aquatic worm blobs has yielded significant insights for the field of soft matter physics, particularly in the emerging area of “topologically entangled living polymers”. The concept of a topologically non-equilibrium “living” polymer^125^ has been extensively explored, particularly in the context of surfactant and DNA-enzyme mixtures, such as topoisomerase.^126–129^ These systems leverage a diverse range of proteins capable of precisely regulating DNA topology, providing exciting possibilities for engineering material properties.

The worm blob, as a living material, expands the definition of living polymers and exemplifies the intricate coupling of bulk mechanics and morphological computation. These active, entangled living polymers, capable of dynamically modulating their rheology and topology, bear striking similarities with synthetic polymer solutions that exhibit tunable physical properties. This positions the worm blobs as a promising experimental platform for probing the physics of out-of-equilibrium polymers.

The worm blob provides a unique opportunity to revisit and redefine the principles of soft matter physics, such as entanglement, reptation, rheological plasticity and elasticity, and fluid-structure interactions, by incorporating activity into these concepts. The initial studies^40,42^ reveal that these worms, unlike traditional polymers, do not exhibit thermal fluctuations but do display randomizing fluctuations. The rheological analysis suggests that, at a first-order approximation, the polymer-like worms behave similarly to classical polymers, with the activity of the worms producing an orientational randomizing effect akin to thermal fluctuations. Anomalies have also been observed in comparison to conventional polymers concerning concentration, which may be attributed to the non-Brownian nature of this system and the friction between the worms. The tunable activity of this system also introduces intriguing aspects, such as variations in the degree of shear-thinning and interactions with the imposed flow. The similarities and differences which have been observed between the system of actively driven polymer-like living worms and well-known polymer solutions experiencing thermal fluctuations warrant further investigation. Moreover, it is crucial to consider the fact that these particles are denser than their surrounding environment and, consequently, subject to gravity. This aspect should also be thoroughly examined in future studies.

The exploration of reversible tangle topology and the development of topological tools for quantifying tangle dynamics can advance our understanding of the worm blob's functionality. This approach has the potential to reshape the field of soft matter physics and provide valuable insights into the behavior of topologically entangled living polymers.

### Towards soft robotic blobs

We envision soft, slender, spaghetti-like filamentous robots that transition topologically from floppy individuals to cohesive, emergent, task-capable soft robotic ensembles. This concept is reminiscent of the science fiction depicted in “The Blob”, and we anticipate that studying actual worm blobs will help turn this concept into reality. Investigating these worms could potentially pave the way for new classes of mechanofunctional active matter systems and collective emergent robotics.

We briefly discuss two swarm robotics examples that exemplify this vision. In the case of small aspect ratios, a ‘robophysical’ analog of the worm blob was recently demonstrated.^23^ This concept involves individual robots comprising six three-link, two-revolute joints with planar, smart, active particles (smarticles) and two light sensors.^24^ This robophysical realization of the blob displays the crawling motion of a biological worm blob by leveraging two major principles: mechanical interactions (entanglements) and function (gait) differentiation. This class of task-capable synthetic systems have also recently been described as ‘amorphous entangled active matter’.^116^

In a second example, another group utilized large aspect ratio, slender and soft actuators that employ entanglement to hold soft materials.^25^ It involved the use of fluidically actuated slender hollow elastomeric filaments that would coil up and entangle around the object being gripped. One can imagine robots similar to these forming entangled blobs and actuating spontaneous tangling and untangling in a similar fashion as the actual worm blob. It would be interesting to investigate the possibility of locomotion and topological transitions in such slender robot blobs.

Achieving a coherent swarm of slender entangled robots for actuation and manipulation of soft objects has only just begun, as new materials and state-of-the-art controller protocols continue to be developed.^24,25,116,130^ Guided by the Krogh principle^131^ stating that “for such a large number of problems, there will be some animal on which it can be most conveniently studied”, we specifically choose living worms as a model system for studying active polymer physics and the associated emergent collective dynamics in topologically tangled living matter. The worm blob can serve as a guide for designing a decentralized system of soft filamentous robots that can work together. Rather than overlooking topological and steric interactions in robotic design, there is promise in embracing them as a feature for the future of entwined, adaptive, and collective filamentous robotics.

## Author contributions

A. D. and M. S. B. conceptualized this perspective article. All authors contributed to the writing and revision of the manuscript.

## Conflicts of interest

The authors declare no conflicts of interest.

## Supplementary Material
